# Supplementary material for: High sensitivity pressure and temperature quantum sensing in pentacene-doped p-terphenyl single crystals
Source: Nat Commun. 2025 Nov 26;16:10530. doi: 10.1038/s41467-025-65508-2 (PMC12658087; doi:10.1038/s41467-025-65508-2)
Supplement: Supplementary file 1 — Supplementary Information [file 41467_2025_65508_MOESM1_ESM.pdf]

Supplementary Information:
High sensitivity pressure and temperature quantum sensing in pentacene-doped p-terphenyl single crystals

Harpreet Singh,[1,2] Noella D'Souza,[1,3] Joseph Garrett,[1] Angad Singh,[1] Brian Blankenship,[1] Emanuel Druga,[1] Riccardo Montis,[4] Liang Z. Tan,[5] and Ashok Ajoy[1,3,6]

[1] *Department of Chemistry, University of California, Berkeley, Berkeley, CA 94720, USA.*
[2] *Department of Physics, Guru Nanak Dev University, Amritsar, Punjab 143005, India.*
[3] *Chemical Sciences Division, Lawrence Berkeley National Laboratory, Berkeley, CA 94720, USA.*
[4] *Dipartimento di Scienze Pure e Applicate (DiSPEA), Università degli Studi di Urbino Carlo Bo, Urbino, I-61029, Italy.*
[5] *Molecular Foundry, Lawrence Berkeley National Laboratory, Berkeley, CA 94720, USA.*
[6] *CIFAR Azrieli Global Scholars Program, 661 University Ave, Toronto, ON M5G 1M1, Canada.*

Supplementary Note 1. Sample Preparation

The procedure to crystallize pentacene-doped p-terphenyl was adapted from Oxborrow et. al. 2012 [1]. p-terphenyl ($\geq$99.5% purity) and pentacene (99% purity) were purchased from Sigma-Aldrich. To further purify these raw materials, p-terphenyl was loaded into a borosilicate tube (I.D.: 8 mm, O.D.: 10 mm) and sealed under vacuum and inert atmosphere. The tube was then passed through $>$30 rounds of zone refinement. Pentacene was purified via sublimation under continuous Ar (99.999% purity) supply in a borosilicate tube (I.D.: 8 mm, O.D.: 10 mm) in the dark to prevent light-induced disproportionation reactions [2, 3]. Under inert atmosphere, a 1:1000 (w/w) ratio of purified pentacene and p-terphenyl was ground in a mortar and pestle via liquid-assisted grinding with a few drops of toluene and loaded into a homemade borosilicate glass crystal growth ampule (I.D.: 8 mm, O.D.: 10 mm). The ampule was flame-sealed under vacuum and inert atmosphere and loaded in the growth furnace. A preprogrammed stepper motor gradually lowered the ampule through a temperature gradient at 5 mm/hr to produce pentacene-doped p-terphenyl single crystals, following the Bridgman method described in Ref. [4, 5].

Cost Analysis: Here, we detail how we arrived at the $\$2.06$ materials cost per crystal quoted in the main manuscript. Note that all prices quoted are current as of 4/22/2025.

Materials Costs: For a typical PDP crystal, ≈ 1 mg of pentacene is doped into ≈ 1 g of p-terphenyl. Referencing Sigma Aldrich prices of $179 for a 100 gram stock bottle of p-terphenyl and $1350 for a 5 gram stock of pentacene, this comes to $2.06 per ≈ 1g crystal sample ($0.00206 per mg or $0.00000249 per ppb pentacene). NV^- diamond samples are typically purchased from specialized commercial entities, eliminating the need for in-house high cost instrumentation for chemical vapor deposition (CVD), high-pressure high-temperature (HPHT) growth, and beamline work. A representative ThorLabs-supplied, Element Six-fabricated commercial DNVB1 single crystal NV^- diamond sample has 300 ppb NV^- concentration and is sold at $1542.24 ($97.92 per mg or $5.14 per ppb NV^-). The highest concentration DNVB14 diamond sample they supply has a 4.5 ppm NV^- concentration, costing $3325.46 ($211.14 per mg or $0.74 per ppb NV^-).

Fixed Costs Associated with Growth: p-terphenyl and pentacene are purified in-house prior to the crystal growth, so it is useful to consider how this extra purification step contributes in the cost analysis. p-terphenyl is purified via zone-refinement. The setup we use employs nichrome wire, a 24 V power supply, glass tubing, a mechanical shuttling system (actuator and motor), and a motion control programmer. The primary costs here are for the power supply, mechanical actuator, and motion programmer, which cost $52, ≈$270, and $46, respectively.

Pentacene is purified via sublimation, accomplished in a home-built horizontal furnace. This setup consists of wire, a 24 V power supply, glass tubes, and high purity Argon gas. Presuming excess wire and glass tubes

remain from the zone refinement setup construction, the primary cost of this step are the Ar gas cylinder and the power supply: $66 and $52, respectively.

Bridgman crystal growth is done in a home built furnace which uses wire, a ceramic tube, a 24 V power supply, a mechanical shuttling system, and a motion control programmer. The primary costs here are the power supply, shuttling system, and the motion control, $52, ≈$270, and $46 respectively. The total cost, inclusive of preparation and crystal growth supplies, is a fixed ≈$900 addition to the overall material price. Once multiple crystals are produced, the additional cost per crystal is just the material cost ≈$2.

Summary: From a purely materials standpoint, even without normalization, buying the two stock chemicals to make a PDP crystal is cheaper than buying the cheapest ThorLabs-supplied Element Six diamond sample ($1529 vs. $1542). Normalization by mass ($0.00206 per mg PDP vs. $97.92 per mg or $211.14 per mg $NV-$ diamond, depending on the sample) OR by spin density ($0.00000249 per ppb pentacene vs. $5.14 per ppb NV or $0.74 per ppb NV, depending on the sample) further highlights the cost benefits of using PDP crystals, relative to NV centers. The additional cost for the two purification and growth setups is a one-time, initial ≈$900 investment (≈$350 per step) which is still cheaper than commercial chemical vapor deposition instrumentation for diamond fabrication. With all sample production and material costs taken into consideration, we conclude that PDP crystals are a significantly cheaper alternative relative to NV^- diamonds.

Supplementary Note 2. ODMR contrast of T_{yz} and T_{xz}

The steady state populations of T_z, T_y, T_x are 0.08:0.16:0.76 respectively. Supplementary Figure S1A shows the ODMR contrast variation with temperature for the T_{yz} transition, and Supplementary Figure S1B shows the same for the T_{xz} transition. Contrast variation with temperature for the T_{xy} transition is shown in main paper Fig. 2E. At lower temperature (≈ 80K) the T_{yz} contrast changes sign from positive to negative.

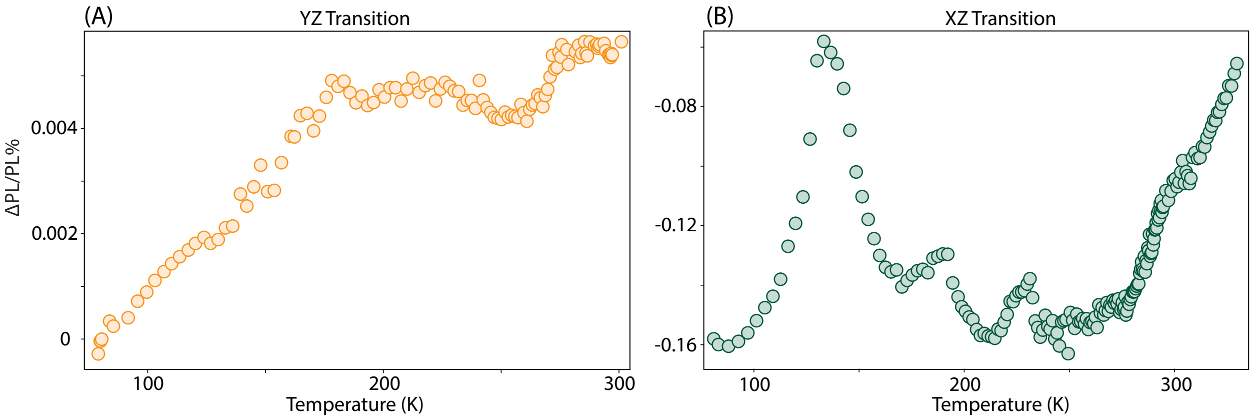

Supplementary Figure S1 ODMR contrast variation with temperature for the (A) T_{yz} transition and (B) T_{xz} transition.

Supplementary Note 3. Sensitivity Measurement

Temperature sensitivity estimation: As mentioned in the main text, to evaluate the time-normalized temperature sensitivity of our measurements, we use $\eta^T = \sigma\sqrt{\tau}/\frac{dS}{dT}$[6], where $\frac{dS}{dT}$ is the maximum ODMR signal slope with temperature, σ is the noise floor (9.6×10^{-6}, 13.5×10^{-6}, and 19.5×10^{-6} for T_{xy}, T_{yz}, and T_{xz} respectively), and τ is the integration time (300 ms for our experiments), defined by the low-pass filter's settling time in the detection lock-in amplifier. Supplementary Figure S2 shows the signal variation with temperature at resonance frequency 101.6 MHz, 1344.2 MHz, and 1443.4 MHz.

Our setup is not optimized for sensitivity; we collect only a small fraction of photons, and the ODMR contrast in

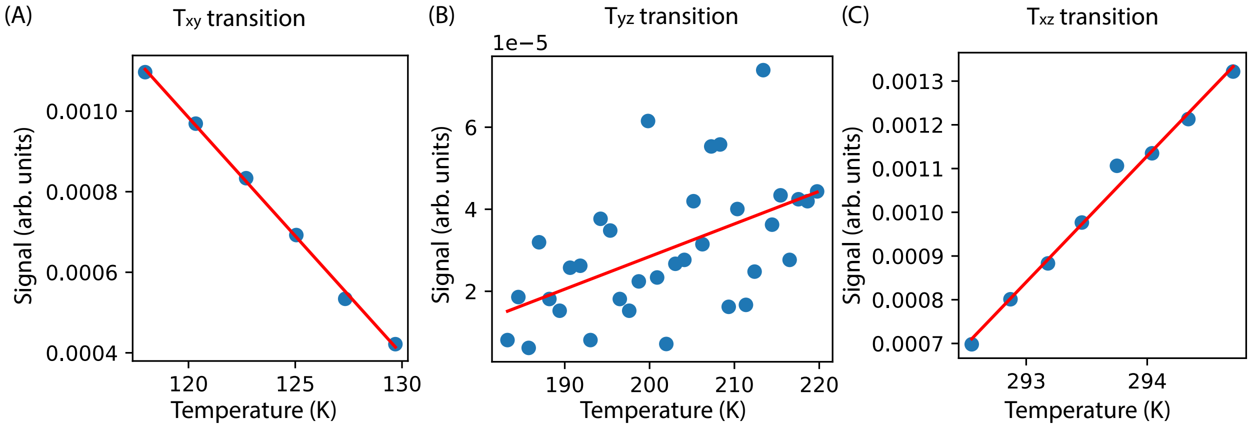

Supplementary Figure S2 Signal variation vs temperature (A) T_{xy}, (B) T_{yz}, and (C) T_{xz} transitions.

main text Fig. 2E is low due to continuous-wave, instead of pulsed, illumination. Both factors could be improved by at least an order of magnitude [7].

Pressure sensitivity estimation: Pressure sensitivity can be evaluated as $\eta^P = \sigma\sqrt{\tau}/\frac{dS}{dP}$, and is reported in Supplementary Table S1. The T_{xy} transition ($2E$, where E is the transverse zero-field splitting parameter) is not employed for pressure sensing due to its low sensitivity to pressure (see Supplementary Figure S4). Supplementary Figure S3 shows the signal variation with pressure at a frequency of 1327.5 MHz (1437.5 MHz) for T_{yz} (T_{xz}). The estimated σ is 17.5×10^{-6} (4.8×10^{-6}) for T_{yz} (T_{xz}). Even without optimization, the pressure sensitivity for PDP (≈0.07Bar/$\sqrt{\mathrm{Hz}}$).

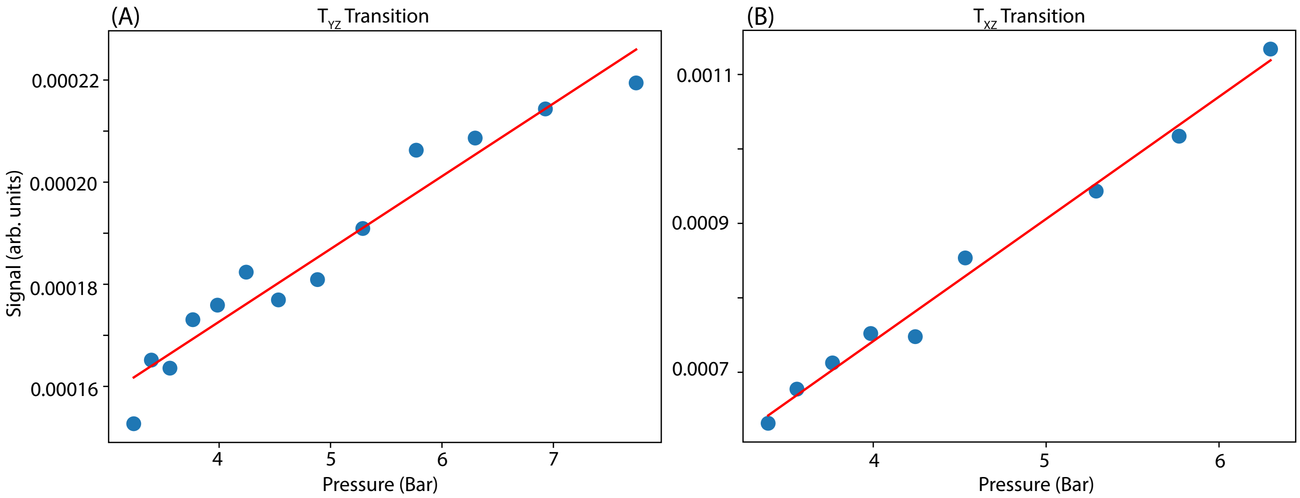

Supplementary Figure S3 Signal variation vs pressure (A) T_{yz} transition (B) T_{xz} transition.

Supplementary Note 4. Expanded Sensitivity Table

Supplementary Table S1 is an expanded version of main text Table I. This expanded table contains values for all three pentacene transitions, including values for different temperature or pressure ranges as applicable.

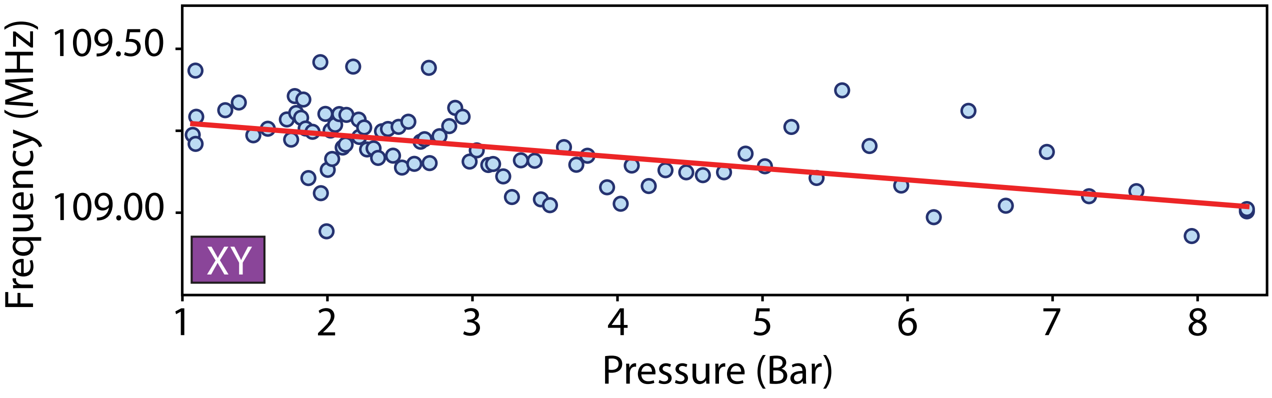

Supplementary Figure S4 T_{xy} transition frequency ($2E$, where E is the transverse zero-field splitting parameter) variation vs pressure Low pressure sensitivity resulted in this transition not being used for pressure sensing.

Material		Linewidth (FWHM)	Max Contrast	Laser Power	Collection Efficiency	Defect Concentration	$\frac{df}{dT}$	η^T	$\frac{df}{dP}$	η^P
		MHz	%	mW	%	ppm	$\frac{kHz}{K}$	$\frac{K}{\sqrt{Hz}}$	$\frac{kHz}{Bar}$	$\frac{Bar}{\sqrt{Hz}}$
Pentacene	XY	4.8	16.8[8]	110	6.94	~1000	6.8[a]<br>247[b]<br>8.7[c]	0.09	NR	NR
	YZ	6.1	NR[8]	110	6.94	~1000	90	9.3	1400†<br>362‡	0.19
	XZ	4.3	NR[8]	110	6.94	~1000	101	0.04	1800†<br>350‡	0.07
NV^-		3.3[9]	30[10]	150[9]	2[12]	<1[12]	74.2[9]	7.6×10^{-4}[11]	1.46[12]	6[12]
V^-_{Si} (SiC)[13]		100	0.11	76	NR	1.6[14]	1100	1	0.031[14]	NR[14]
hBN[11]		34[15]	0.1	<100	1	10.6	684	3.82	91(σ_z)	26.2(σ_z)

a. For 77-125 C, b. For 125-150 C (phase transition region), c. For 150-330oC, † For 1-2 bar, ‡ For 3-8 bar. Ref: [7] Singh et al. Phys. Rev. Research 7, 013192 (2025), measured under pulsed laser excitation. [8] Acosta et al. Phys. Rev. Lett. 104, 070801 (2010), dD/dT cited for diamond. [9] Ho et al. Functional Diamond 1, 160 (2021), [10] Gottscholl et al. Nature Comm. 12, 4480 (2021), [11] Doherty et al. Phys. Rev. Lett. 112, 047601 (2014), dD/dP cited. [12] Kraus et al. Scientific Reports 4, 5303 (2014), dD/dT cited. [13] Wang et al. Nature Materials 22, 489 (2023), dD/dP cited. [14] Stern et al. Nature Comm. 13, 618 (2022).

Supplementary Table S1 Comparison of quantum sensor platforms for temperature and pressure sensing including all pentacene transitions. Source references are shown as footnotes. Slopes of ODMR frequency variations df/dT and df/dP are material properties; while sensitivity values η^T, η^P depend on the measurement specifics. For pentacene, sensitivity values are under currently demonstrated conditions. The first two columns shown ODMR linewidth and contrast but are not employed for sensitivity estimation.

Supplementary Note 5. Luminescence thermometry

Sample luminescence (PL) changes as a function of temperature (see Fig. S5). As the intersystem crossing (ISC) rate decreases with decreasing temperature, a higher PL is observed at lower temperatures. This is because most of the pentacene molecules undergo spontaneous emission from the excited singlet state to the ground singlet state, rather than undergoing ISC into the triplet state. Below, we have plotted the PL vs.

temperature graph and estimated the relative thermal sensitivity, $S_r(T) = 0.11\%$, using the expression:

$$S_r(T) = \left(\frac{1}{PL(T)}\right)\left(\frac{dPL(T)}{dT}\right) \times 100\%.$$

The deviations from linearity could be due to fluctuations in laser power. The sensing experiments presented in the main paper rely on changes in the resonance frequency and signal, which constitute a more robust method, as the resonant frequency is independent of laser power fluctuations.

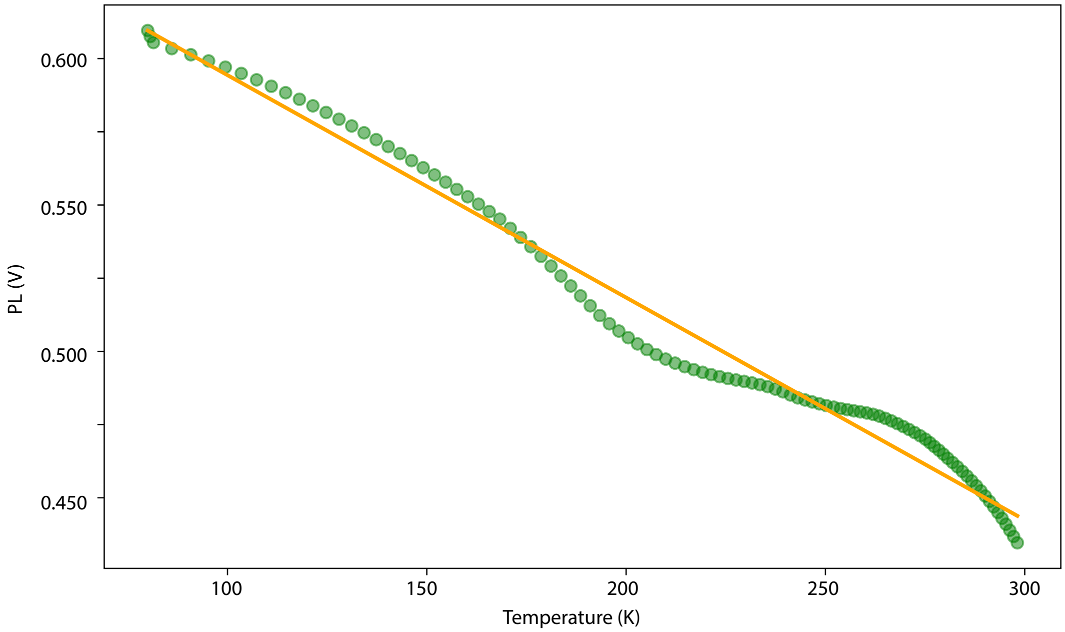

Supplementary Figure S5 Photoluminescence vs temperature

[1] M. Oxborrow, J. D. Breeze, and N. M. Alford, Room-temperature solid-state maser, Nature **488**, 353 (2012).

[2] S. S. Zade, N. Zamoshchik, A. R. Reddy, G. Fridman-Marueli, D. Sheberla, and M. Bendikov, Products and mechanism of acene dimerization. a computational study, Journal of the American Chemical Society **133**, 10803 (2011).

[3] L. B. Roberson, J. Kowalik, L. M. Tolbert, C. Kloc, R. Zeis, X. Chi, R. Fleming, and C. Wilkins, Pentacene disproportionation during sublimation for field-effect transistors, Journal of the American Chemical Society **127**, 3069 (2005).

[4] S. Cui, Y. Liu, G. Li, Q. Han, C. Ge, L. Zhang, Q. Guo, X. Ye, and X. Tao, Growth regulation of pentacene-doped p-terphenyl crystals on their physical properties for promising maser gain medium, Crystal Growth & Design **20**, 783 (2020).

[5] Q. Ai, P. Chen, Y. Feng, and Y. Xu, Growth of pentacene-doped p-terphenyl crystals by vertical bridgman technique and doping effect on their characterization, Crystal Growth & Design **17**, 2473 (2017).

[6] S. Choe, J. Yoon, M. Lee, J. Oh, D. Lee, H. Kang, C.-H. Lee, and D. Lee, Precise temperature sensing with nanoscale thermal sensors based on diamond nv centers, Current Applied Physics **18**, 1066 (2018).

[7] H. Singh, N. D'Souza, K. Zhong, E. Druga, J. Oshiro, B. Blankenship, R. Montis, J. A. Reimer, J. D. Breeze, and A. Ajoy, Room-temperature quantum sensing with photoexcited triplet electrons in organic crystals, Phys. Rev. Res. **7**, 013192 (2025).

[8] V. M. Acosta, E. Bauch, M. P. Ledbetter, A. Waxman, L.-S. Bouchard, and D. Budker, Temperature dependence of the nitrogen-vacancy magnetic resonance in diamond, Physical review letters **104**, 070801 (2010).

[9] K. O. Ho, Y. Shen, Y. Y. Pang, W. K. Leung, N. Zhao, and S. Y. and, Diamond quantum sensors: from physics to applications on condensed matter research, Functional Diamond **1**, 160 (2021), https://doi.org/10.1080/26941112.2021.1964926.

[10] A. Gottscholl, M. Diez, V. Soltamov, C. Kasper, D. Krauße, A. Sperlich, M. Kianinia, C. Bradac, I. Aharonovich, and V. Dyakonov, Spin defects in hbn as promising temperature, pressure and magnetic field quantum sensors, Nature communications **12**, 4480 (2021).

[11] M. W. Doherty, V. V. Struzhkin, D. A. Simpson, L. P. McGuinness, Y. Meng, A. Stacey, T. J. Karle, R. J. Hemley, N. B. Manson, L. C. Hollenberg, *et al.*, Electronic properties and metrology applications of the diamond nv- center under pressure, Physical review letters **112**, 047601 (2014).

[12] H. Kraus, V. Soltamov, F. Fuchs, D. Simin, A. Sperlich, P. Baranov, G. Astakhov, and V. Dyakonov, Magnetic field and temperature sensing with atomic-scale spin defects in silicon carbide, Scientific reports **4**, 5303 (2014).

[13] J.-F. Wang, L. Liu, X.-D. Liu, Q. Li, J.-M. Cui, D.-F. Zhou, J.-Y. Zhou, Y. Wei, H.-A. Xu, W. Xu, W.-X. Lin, J.-W. Yan, Z.-X. He, Z.-H. Liu, Z.-H. Hao, H.-O. Li, W. Liu, J.-S. Xu, E. Gregoryanz, C.-F. Li, and G.-C. Guo, Magnetic detection under high pressures using designed silicon vacancy centres in silicon carbide, Nature Materials **22**, 489 (2023).

[14] H. L. Stern, Q. Gu, J. Jarman, S. Eizagirre Barker, N. Mendelson, D. Chugh, S. Schott, H. H. Tan, H. Sirringhaus, I. Aharonovich, and M. Atatüre, engRoom-temperature optically detected magnetic resonance of single defects in hexagonal boron nitride., Nature communications **13**, 618 (2022), place: England.
